# The origin and current situation of *Fusarium oxysporum* f. sp. *cubense* tropical race 4 in Israel and the Middle East

**DOI:** 10.1038/s41598-020-58378-9

**Published:** 2020-01-31

**Authors:** Marcel Maymon, Noa Sela, Uri Shpatz, Navot Galpaz, Stanley Freeman

**Affiliations:** 1 Department of Plant Pathology and Weed Research, ARO, The Volcani Center, Rishon LeZion, 7505101 Israel; 2 Department of Microbiology and Plant Pathology, The Robert H. Smith Faculty of Agriculture, Food and Environment, The Hebrew University of Jerusalem, Rehovot, 7610001 Israel; 3 Northern R & D, Kiryat Shmona, 11016 Israel

**Keywords:** Pathogens, Plant sciences

## Abstract

*Fusarium oxysporum* f.sp. *cubense* (Foc) is considered one of the most devastating soilborne fungal pathogens of banana worldwide. Foc causing mortality to Cavendish group bananas, and belonging to the unique vegetative compatibility group (VCG) 01213/16 has been termed tropical race 4 (TR4) and has currently been renamed *F. odoratissimum*. The pathogen that was first detected approximately 50 years ago in South East Asia, has since spread to countries within the greater Mekong subregion and to Australia. Recently, the pathogen disseminated to India, Pakistan, Oman and Mozambique (Africa) and was identified in the South American continent in Colombia in 2019. In the Middle East, TR4 was first reported from Jordan and Lebanon, and later from Israel in 2016. In Israel, the pathogen was identified as TR4 by VCG tests, pathogenicity assays and molecular verification. The complete genomes of five representative TR4 isolates including two from Israel, one from Jordan, one from the Philippines, and one from Indonesia were sequenced, and single nucleotide polymorphisms (SNPs) analyses were conducted. SNPs were compared to 11 additional sequenced TR4 isolates, to determine the origin of the Israeli isolates. SNP detection and phylogeographical analyses determined that the Middle Eastern isolates are closely related, indicating that the pathogen most likely spread to Israel from Jordan, while those from Colombia are related to a representative isolate from Indonesia.

## Introduction

Banana (*Musa* spp.) is one of the most popular exported fruit and serves as a staple diet for millions of people worldwide^1^. In 2017, the approximate volume of global production reached 114 million tons totaling a gross value of about US$8 billion per year^2^. An estimated 15% of all production reaches international markets, most of which are from cultivars within the Cavendish subgroup. Cavendish cultivars are the most popularly grown and exported bananas worldwide today, due to their resistance to ‘Panama disease’ caused by the soil-borne fungus *Fusarium oxysporum* f.sp. *cubense* (Foc) race 1, that eliminated the susceptible ‘Gros Michel’ cultivar and related industry^1,3^.

‘Panama disease’ or Fusarium wilt, caused by *F. o*. f.sp. *cubense* (Foc) race 1, was first described in Australia in 1874^4^. The pathogen destroyed the well-established ‘Gros Michel’ cultivar banana industry that was grown in monoculture plantations in the Americas, Africa and in the Far East during the 1900s^5^. Thus, Fusarium wilt became known as a pathogen of significant global importance. The cultivar ‘Gros Michel’ was subsequently replaced by resistant Cavendish group cultivars, however, the disease recurred approximately 50 years ago in the 1970’s in the southeast Asian continent and Australia with the detection of a new race of Foc, tropical race 4 (Foc TR4), causing mortality to Cavendish cultivars^6^. Since then the disease has spread throughout South-East Asia, to the Middle-East, India and Pakistan, and was recently discovered in the African continent in Mozambique in 2015^7,8^. Foc TR4 was first detected in the south East Asian subcontinent of Taiwan in 1967, after most likely being introduced on infected plants from Indonesia^9,10^. The pathogen was then disseminated into the Chinese provinces of Fujian, Guangdong, Guangxi, Hainan and Yunnan^11–14^. The distribution of infected planting material and heavy farming equipment was probably the cause of further dissemination of the pathogen into the neighboring countries of Laos, Myanmar, Vietnam, and Cambodia^8^. The recent report of spread of TR4 into the Indian subcontinent is of major concern since India is the largest producer of bananas worldwide^15^. Approximately 70% production is of the Cavendish cultivar and the fruit is widely consumed and regarded as a staple diet^5^. However, the recent detection of TR4 in four plantations in the north of Colombia, published by García-Bastidas *et al*.^16^ and renamed as *Fusarium odoratissimum*, will likely have devastating consequences to the banana industry and exports worldwide.

**Figure 1:**
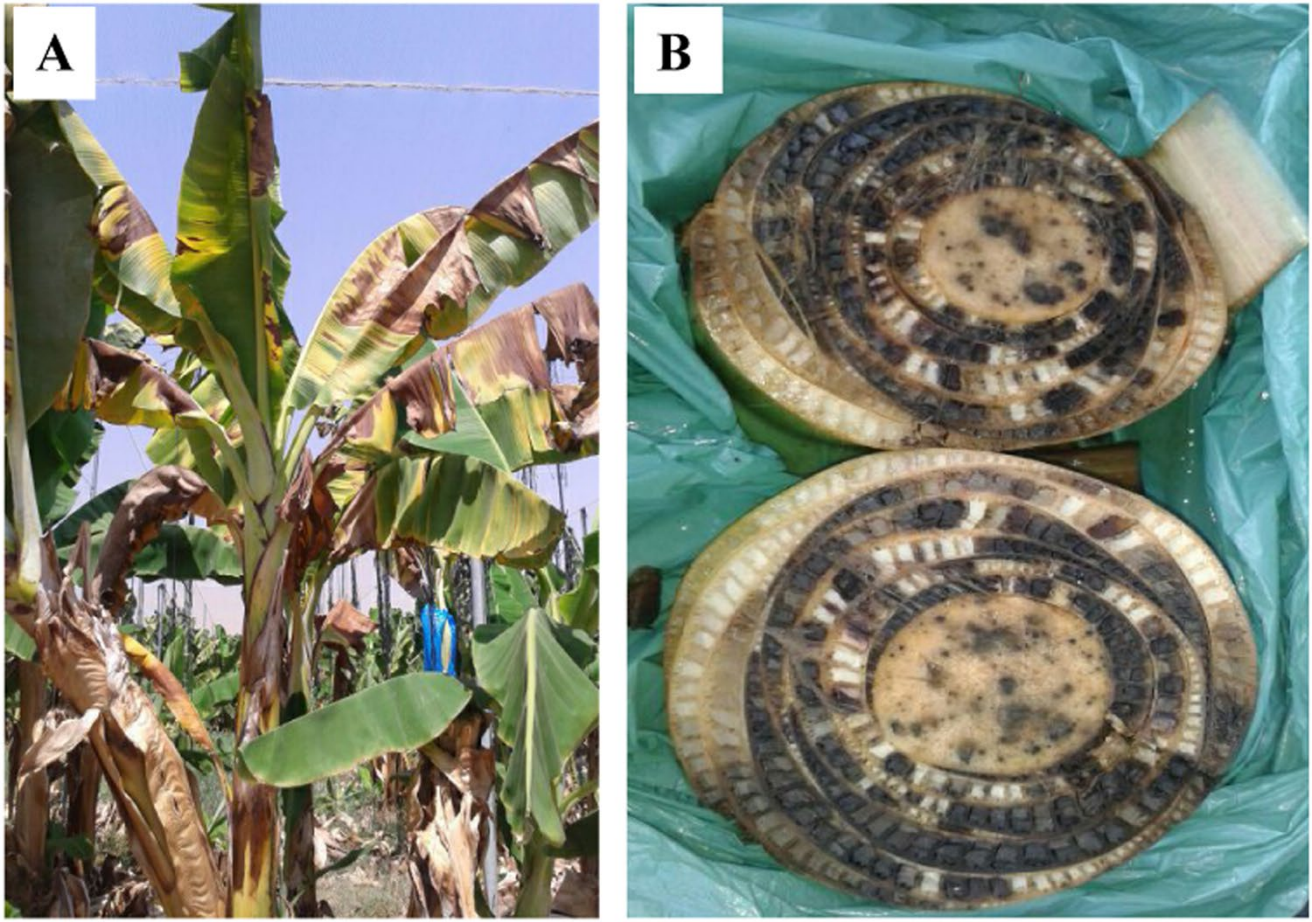
Typical external symptoms of Fusarium wilt of Cavendish banana (cv. ‘Grande Naine’) caused by *Fusarium oxysporum* f. sp. *cubense* (race TR4) in an infected plantation at Shfeya, Carmel coastal plain, Israel (**A**). Cross section of infected vasculature of infected xylem vessels (**B**).

In the Middle East, Foc TR4 has been reported from Lebanon^17^, Jordan^18^ and most recently from Israel^19^. In certain areas, TR4-infected bananas in Jordan are cultivated in very close proximity (1–10 km) to those of infected banana plots in Israel^3^. An additional banana cultivation area of 200 ha in Jericho, within the Palestinian Authority, is a region that also needs to be re-evaluated for the presence/absence of disease^3^. In Israel, Cavendish bananas are cultivated in the lower Carmel coastal plain (1200 ha.), western Galilee (600 ha.), western Negev (100 ha.) and Jordan valley (850 ha.). During July 2016, typical Fusarium wilt symptoms were observed in mature ‘Grande Naine’ Cavendish plants from Shfeya, Carmel coastal plain and 2 months later, in plants from Kibbutz Ein Gev, eastern shore of Lake Galilee^19,20^ (Fig. 1).

At the time, routine surveys in banana fields adjacent to infected areas, did not reveal any new cases of disease. However, during the summer and autumn of 2018, intensive surveillance operations discovered an additional outbreak in a number of plots close to some of the original locations (in the eastern/southern Lake Galilee area)^20,21^. As in previous cases, the infected sites were confined and placed under strict supervision of the Israeli NPPO. The affected plantations were fenced off, access restricted and entry allowed under strict quarantine conditions only. The spread of fungal spores in rainwater was restricted by ditches that were dug around fenced affected areas. The status of *Fusarium oxysporum* f. sp. *cubense* TR4 in Israel has been declared officially by the NPPO as: “actionable, under eradication”^21^.

According to a study by Zheng *et al*.^8^, a combination of classical morphological identification, phenotyping assays and sequence analyses revealed a very close relationship between the Foc TR4 isolates in the Greater Mekong Subregion (GMS), where intensive banana cultivation is conducted. Analyses of SNPs (Single Nucleotide Polymorphisms) allowed the researchers to determine the phylogeography of Foc TR4 across the GMS, Indian subcontinent, and certain areas in the Middle East, revealing three distinct Foc TR4 sub-lineages^8^. Genetic diversity of Foc populations can be determined by vegetative compatibility groupings (VCGs)^22^. Four representative groups of Foc were used to differentiate populations within a defined race lineage^5^. TR4 populations from different worldwide regions indicate that the pathogen is clonal and belongs to a single VCG 01213–01216 complex, thus serving as a reliable indicator for the presence of this specific pathogen^3,5^. Likewise, DNA molecular analyses including species-specific primer amplification and sequence analyses have been used reliably for the identification and verification of TR4 isolates from various affected areas^23,24^.

The Foc pathogens act as typical soilborne fungi. Resting spores of the pathogen can remain viable in soil for decades, thus limiting the cultivation of susceptible banana germplasm in infested soils^1,3^. The fungus penetrates the roots spreading to the vasculature, releasing toxins, and causing plants to secrete gelatinous substances that eventually causes wilting and mortality of the banana plant^25^. Vegetative propagation of planting material and reliance on nearly exclusively based ‘Cavendish’ lines that are susceptible to TR4 has threatened the production of export and local banana industries. Thus, prevention and management strategies at national and international levels must be implemented against this destructive disease that seriously threatens the global banana industry^26,27^. To date, no effective TR4 management methods are known and no alternative commercial banana clones resistant to the pathogen exist^3^, although transgenic clones transformed with a gene from TR4-resistant diploid bananas and a nematode-derived antiapoptosis gene remain disease free^28^, while silencing of vital fungal genes^29^ have been shown to confer efficient resistance against disease. Disease management has proven to be difficult, therefore, prevention is currently the main strategy to avoid new Foc TR4 incursions^3,6^.

In this study, we report on the presence and origin of Foc TR4 in Cavendish plantations in Israel and the Middle East. The main aims of this research were to determine the: 1) genetic diversity of the TR4 FOC isolates in Israel compared with representative worldwide isolates, and 2) geographic origin of the population in Israel using genome sequencing data, towards understanding the epidemiological impact of spread of the pathogen within the Middle East.

## Results

### VCG characterization of isolates

VCG analysis was carried out using nit mutants produced from diagnostic isolates, paired with tester Nit M mutants generated in this research and with representative isolates obtained from A. Viljoen (Table 1). Heterokaryons were usually evident within 10 to 15 days. When mutants formed a prototrophic heterokaryon, their parent isolates were assigned to the same VCG. VCG analysis of monoconidial *F. oxysporum* isolates recovered from infected samples in Israel (FOC TR4-1, FOC TR4-5, FOC TR4-16, FOC TR4-18, FOC TR4-26, FOC TR4-27) compared with representative TR4 isolates (II5 from Indonesia, S1B8 from the Philippines and JV11 from Jordan), confirmed the presence of TR4 in Israel. Nit mutants from the diagnostic isolates anastomosed to form stable heterokaryons when paired with the TR4 Nit M testers on minimal media, thus confirming their identity as VCG 01213/16, unique for TR4. Representative isolates of different VCGs (CAV 095 and CAV 105 belonging to VCG 0120; CAV 188 belonging to VCG 1212 and CAV 786 belonging to VCG 0124) did not anastomose with the unique TR4 representative VCG 01213/16 isolates, further indicating reliability of the assay.

**Table 1:** Isolates of *Fusarium oxysporum* f. sp. *cubense* used in this study. *Complete genomes sequenced in this study. **Sequenced by Zheng *et al*.^8^. ***Nd - Not determined; ****Sequenced by García-Bastidas *et al*.^16^. *****Kindly provided by R. Ploetz^19^. ******Kindly provided by A. Viljoen^36^.

Designation	Date sampled	Origin	VCG grouping
*FOC TR4-1	08.2016	Shfeya, Israel	01213/16
FOC TR4-2	08.2016	Shfeya, Israel	01213/16
FOC TR4-3	08.2016	Shfeya, Israel	01213/16
FOC TR4-4	08.2016	Shfeya, Israel	01213/16
*FOC TR4-5	08.2016	Ein Gev, Israel	01213/16
FOC TR4-6	08.2016	Ein Gev, Israel	01213/16
FOC TR4-7	08.2016	Ein Gev, Israel	01213/16
FOC TR4-8	08.2016	Shfeya, Israel	01213/16
FOC TR4-9	08.2016	Shfeya, Israel	01213/16
FOC TR4-10	08.2016	Shfeya, Israel	01213/16
FOC TR4-11	08.2016	Shfeya, Israel	***Nd
FOC TR4-12	08.2016	Shfeya, Israel	***Nd
FOC TR4-13	09.2018	Masada, Israel	***Nd
FOC TR4-14	09.2018	Masada, Israel	***Nd
FOC TR4-15	09.2018	Masada, Israel	***Nd
FOC TR4-16	09.2018	Masada, Israel	01213/16
FOC TR4-17	09.2018	Masada, Israel	***Nd
FOC TR4-18	10.2018	Gesher, Israel	01213/16
FOC TR4-19	10.2018	Masada, Israel	***Nd
FOC TR4-20	10.2018	Masada, Israel	***Nd
FOC TR4-21	10.2018	Masada, Israel	***Nd
FOC TR4-22	10.2018	Masada, Israel	***Nd
FOC TR4-23	10.2018	Masada, Israel	***Nd
FOC TR4-24	10.2018	Masada, Israel	***Nd
FOC TR4-25	10.2018	Gesher, Israel	***Nd
FOC TR4-26	10.2018	Degania B, Israel	01213/16
FOC TR4-27	10.2018	Degania B, Israel	01213/16
FOC TR4-28	12.2018	Gesher, Israel	***Nd
FOC TR4-29	12.2018	Gesher, Israel	***Nd
FOC TR4-30	12.2018	Gesher, Israel	***Nd
FOC TR4-31	12.2018	Gesher, Israel	***Nd
FOC TR4-32	12.2018	Gesher, Israel	***Nd
FOC TR4-33	12.2018	Degania B, Israel	***Nd
FOC TR4-34	12.2018	Degania B, Israel	***Nd
FOC TR4-35	12.2018	Degania B, Israel	***Nd
FOC TR4-36	12.2018	Degania B, Israel	***Nd
FOC TR4-37	12.2018	Degania B, Israel	***Nd
*II-5	Unknown	Indonesia (Ploetz)	*****01213/16
*S1B8	Unknown	Philippines (Ploetz)	*****01213/16
**JV11	Unknown	Jordan (Ploetz)	*****01213/16
*JV14	Unknown	Jordan (Ploetz)	*****01213/16
**Phi2.6 C	Unknown	Philippines [Zheng *et al*. (2018)]	***Nd
**Pak1.1 A	Unknown	Pakistan [Zheng *et al*. (2018)]	***Nd
**VN-2	Unknown	Vietnam [Zheng *et al*. (2018)]	***Nd
**My-1	Unknown	Myanmar [Zheng *et al*. (2018)]	***Nd
**La-2	Unknown	Laos [Zheng *et al*. (2018)]	***Nd
**Leb1.2 C	Unknown	Lebanon [Zheng *et al*. (2018)]	***Nd
****Col 2	06.2019	Colombia [García-Bastidas *et al*. (2019)]	01213/16
****Col 4	06.2019	Colombia [García-Bastidas *et al*. (2019)]	01213/16
****Col 17	06.2019	Colombia [García-Bastidas *et al*. (2019)]	01213/16
CAV-095	Unknown	Kiepersol, South Africa (Viljoen)	******0120
CAV-105	Unknown	Kiepersol, South Africa (Viljoen)	******0120
CAV-188	Unknown	Tanzania (Viljoen)	******01212
CAV-786	Unknown	Australia (Viljoen)	******0124

### Molecular characterization of isolates by ap-PCR and specific PCR analyses

**Figure 2:**
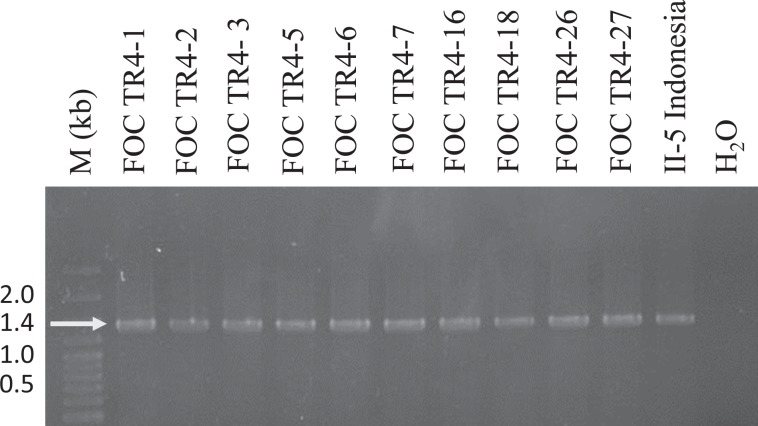
Molecular identification of *Fusarium oxysporum* f. sp. *cubense* tropical race 4 (TR4) of representative isolates from Israel, according to O’Neill *et al*.^24^. A specific DNA band of 1.4 Kb was amplified for TR4 isolates from Israel (FOC TR4-1, FOC TR4-2 and FOC TR4-3 from Shfeya; FOC TR4-5, FOC TR-6, FOC TR4-7 from Ein Gev; FOC TR4-16 from Masada; FOC TR4-18 from Gesher; FOC TR4-26 and FOC TR4-27 from Deganya B), representative TR4 isolate from Indonesia (II-5) and water control (H_2_O). M: DNA marker with sizes in kb.

**Figure 3:**
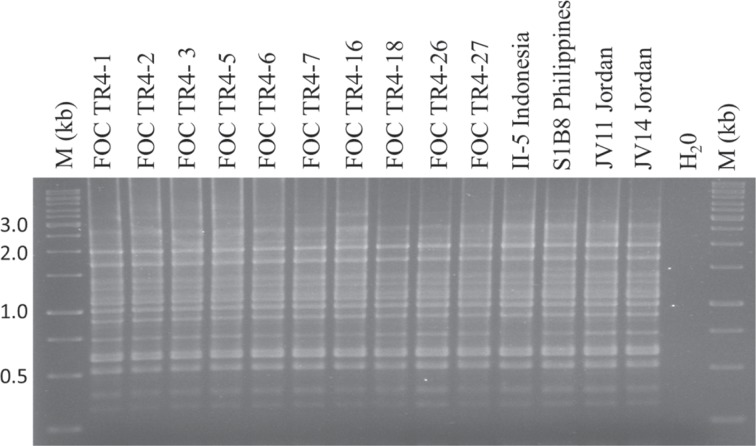
Band patterns of ap-PCR-amplified genomic DNA from *Fusarium oxysporum* f. sp. *cubense* tropical race 4 (TR4) representative isolates from Israel (FOC TR4-1, FOC TR4-2 and TR4-3 from Shfeya; FOC TR4-5, FOC TR4-6, and FOC TR4-7 from Ein Gev; FOC TR4-16 from Masada; FOC TR4-18 from Gesher; FOC TR4-26 and FOC TR-27 from Deganya B), from Indonesia (II-5), from the Philippines (S1B8) and from Jordan (JV11 and JV14), and water control (H_2_O) using primer pair (GACA)_4_. M: DNA markers with sizes in kb.

DNA from representative isolates of *Fusarium oxysporum* f. sp. *cubense* race TR4 and isolates associated with Fusarium wilt of banana in Israel, were amplified with species-specific TR4 primers. An *F. o*. f. sp. *cubense* TR4-specific amplicon of 1.4 kb was amplified from the representative and suspect Israeli isolates (Fig. 2). Genotyping of all 37 TR4 isolates was conducted using ap-PCR with four repeat-motif primers. Among the tested isolates, genetically distinct and identical banding patterns were displayed for all TR4 isolates, as shown with primer (GACA)_4_ (Fig. 3), and primers (CAG)_5_, (AGG)_5_ and (GACAC)_3_ (data not shown).

Thus, specific primer amplification and ap-PCR molecular analyses of all the recovered TR4 isolates from infected samples from Israel and representative isolates from abroad, confirmed the presence of TR4 in Israel (Figs. 2 and 3).

### Pathogenicity assays

**Figure 4:**
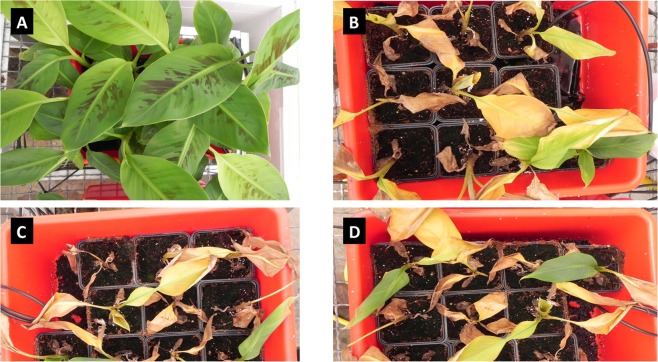
Pathogenicity confirmation assays with selected isolates of *Fusarium oxysporum* f. sp. *cubense* tropical race 4 (TR4), 4 weeks after inoculation of Cavendish plants (cv. ‘Grande Naine’) in Israel. (**A**) water inoculated negative control; (**B**) FOC TR4-2 (from Shfeya, Carmel coastal plain); (**C**) FOC TR4-5 (Ein Gev cultivation plot, eastern shore of lake Galilee); and (**D**), FOC TR4-18 (Masada, Jordan Valley) inoculated, wilted plants.

Two weeks after artificial inoculation with the three selected Israeli TR4 isolates, TR4-treated plants expressed typical external Fusarium wilt symptoms (Fig. 4). The disease progressed steadily and four weeks after inoculation, all TR4-treated plants were killed, while water controls remained healthy (Fig. 4). TR4 identification from symptomatic plants was reconfirmed by PCR (data not shown) and the inoculated FOC isolates were re-isolated from diseased plants verifying Koch postulate assays. TR4 was not isolated from asymptomatic water control plants.

### Sequence analysis of Foc TR4 isolates

**Figure 5:**
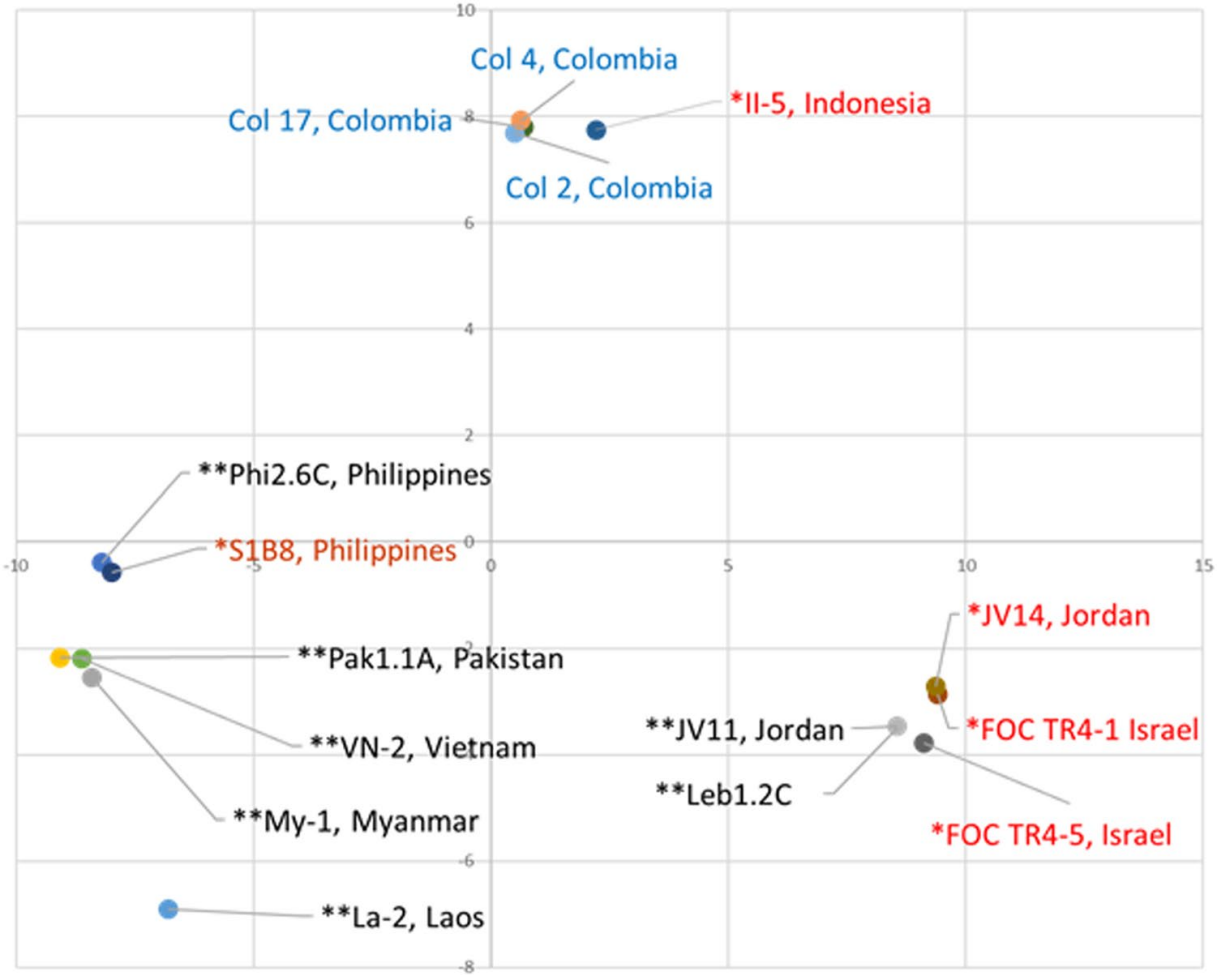
Principal component analysis (PCA) of 15 TR4 isolates of *Fusarium oxysporum* f. sp. *cubense* according to sequenced nuclear polymorphic (SNP) DNAs. PCA was computed by TASSEL software^41^ using SNP data from the complete genomes of five representative isolates that were sequenced in this study (red text): FOC TR4-1 and FOC TR4-5 from Israel; JV14 from Jordan; S1B8 from the Philippines and II-5 from Indonesia, compared to seven isolates sequenced by Zheng *et al*.^8^ (black text): Phi2.6 C from the Philippines; Pak1.1 A from Pakistan; VN-2 from Vietnam; My-1 from Myanmar; La-2 from Laos; JV11 from Jordan; Leb1.2 C from Lebanon, and three isolates Col 2, Col 4 and Col 17 from Colombia, sequenced by García-Bastidas *et al*.^16^ (blue text).

**Figure 6:**
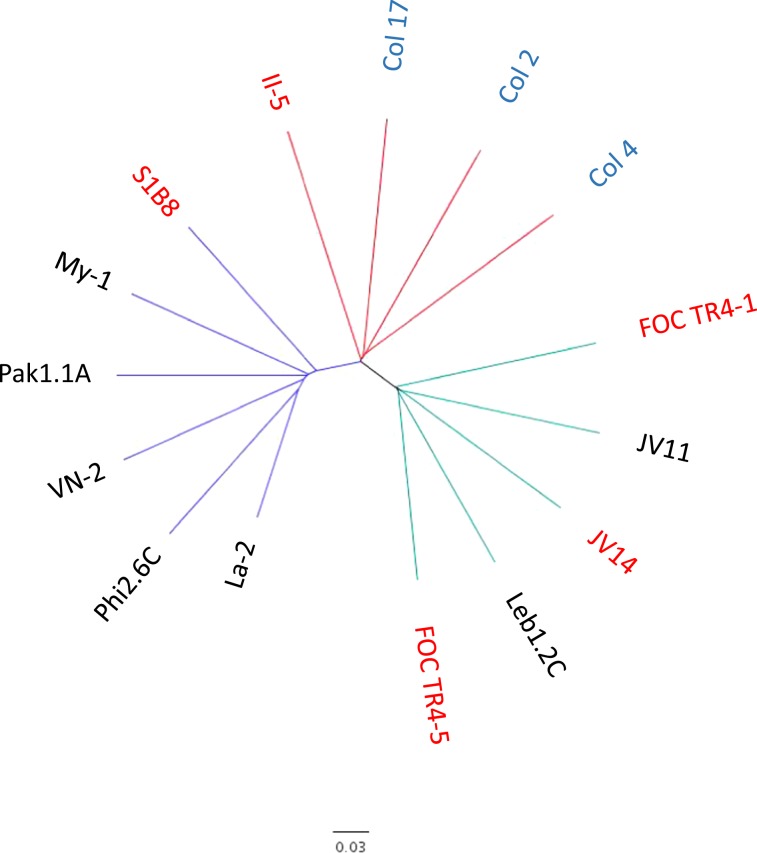
Phylogeny tree of 15 TR4 isolates of *Fusarium oxysporum* f. sp. *cubense* conducted from sequenced nuclear polymorphic (SNPs) found among the different sample DNAs (presented in Fig. 5). The tree which was computed using SNP data by TASSEL software^41^, contains the complete genomes of five representative isolates sequenced in the current study (red text): FOC TR4-1 and FOC TR4-5 from Israel, JV14 from Jordan, S1B8 from the Philippines, and II-5 from Indonesia, compared to seven isolates sequenced by Zheng *et al*.^8^ (black text): Phi2.6 C from the Philippines, Pak1.1 A from Pakistan, VN-2 from Vietnam, My-1 from Myanmar, La-2 from Laos, JV11 from Jordan and Leb1.2 C from Lebanon, and three isolates Col 2, Col 4 and Col 17 from Colombia, sequenced by García-Bastidas *et al*.^16^ (blue text).

**Figure 7:**
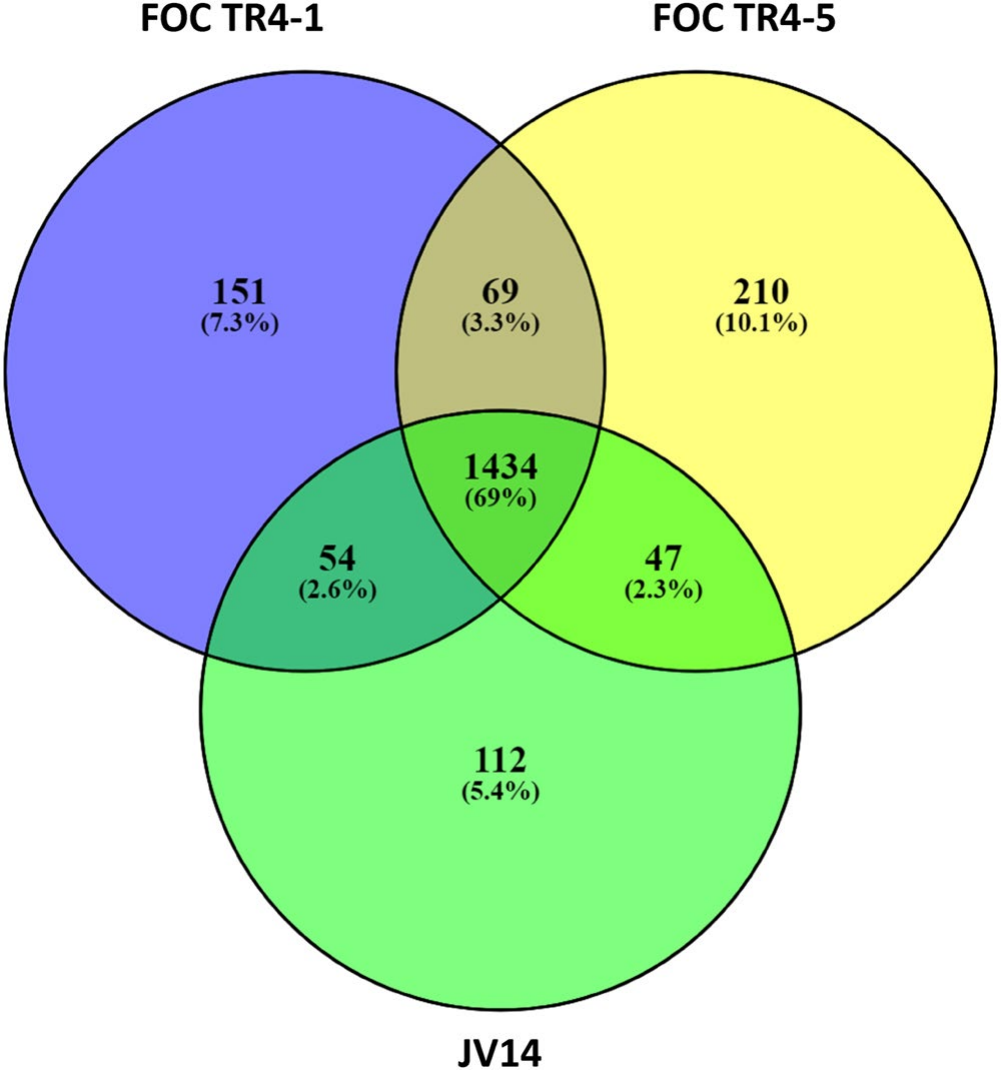
Venn diagram of SNP data from 3 TR4 isolates of *Fusarium oxysporum* f. sp. *cubense* compared to TR4 II5 reference genome sequence (GenBank accession GCA_000260195) FOC TR4-1, FOC TR4-5 from Israel and JV14 from Jordan. Venn created with Venny 2.1 Oliveros, J.C. (2007–2015) Venny, an interactive tool for comparing lists with Venn’s diagrams. https://bioinfogp.cnb.csic.es/tools/venny/index.html.

Whole genome sequencing of representative TR4 isolates from Israel and Jordan and other representative isolates from south East Asia were performed (our sequences have been uploaded to SRA under BioProject accession PRJNA563197), in order to study their genetic relatedness and determine the origin of Israeli isolates. The maximum-likelihood phylogeny of the genome sequences clearly confirmed that these isolates belong to the TR4 genetic lineage (Zheng *et al*.^8^). Single nucleotide polymorphisms (SNPs) of the TR4 isolates from Israel (FOC TR4-1 from Shfeya and FOC TR4-5 from Ein Gev), Jordan (JV14), the Philippines (S1B8), and Indonesia (II-5) were analyzed. These isolates were compared to ten additional isolates sequenced by Zheng *et al*.^8^ and García-Bastidas *et al*.^16^. Subsequent principal component analyses (PCA) and hierarchical clustering revealed three distinct geographical groups of the TR4 isolates: (i) a Middle Eastern clade containing isolates from Israel, Lebanon and Jordan; (ii) a South East Asian clade containing isolates from Laos, Myanmar, Pakistan, Philippines, and Vietnam, and (iii) a clade containing the representative Indonesian isolate and those from Colombia (Figs. 5 and 6). Likewise, five SIX-gene homologues (SIX1a, SIX1b, SIX1c, SIX4, and SIX9) of TR4 isolates based on the consensus sequence showed no differences in their sequences to those of our sequenced isolates (Fig. S1). The Jordanian and Israeli isolates showed a greater common SNP uniformity, as can be seen in the Venn diagram (Fig. 7).

## Discussion

In recent years, the occurrence and spread of *Fusarium oxysporum* f. sp. *cubense* race TR4 has created huge concern within the banana research, cultivation and industry communities. Examples are widespread, from a local incursion in the Northern Territory of Australia, the pathogen reached Northern Queensland where biosecurity officials were unfortunately unable to curb its initial establishment and consequent spread within the major banana cultivation area of the country^24^. Likewise, concern has surmounted due to continuous dissemination of TR4 within the greater Mekong sub-region and suspect origin of the pathogen associated with the involvement of Chinese entrepreneurs in this area^8^. Furthermore, the recent widespread detection of the pathogen in India, the largest banana producer worldwide^15^, and establishment of the pathogen in Africa^7^ and the Middle East^18–20^ has caused much alarm. Of utmost concern is the arrival of TR4 to the continent of America, where numerous reports have stated the presence of the pathogen in the most populous banana cultivation area of Colombia, specifically the northern coastal region of La Guajira^16^.

The first detection of *F. o*. f.sp. *cubense* isolate TR4 in Israel occurred in the summer of 2016 at two limited locations^19^. However, the pathogen has since been detected from at least five additional plots, located in the Jordan Valley, south of the original affected site^20,21^. This is the first comprehensive study pertaining to the identity, occurrence and spread of the pathogen in Israel. Detailed biological, pathological and molecular analyses were conducted in this study on the TR4 isolates from Israel to verify their identity and clonality. Vegetative compatibility groupings of TR4 has reliably allowed an accurate identity of the pathogen since only a single VCG (01213/16) for this race has been identified to date, indicating its uniformity^24^. VCG analyses conducted with 14 Israeli representative Foc TR4 isolates from four different geographic locations in Israel compared with four representative isolates from the Philippines, Jordan and Indonesia, confirmed and corroborated presence of the unique VCG (Table 1) in the Middle East.

Additional molecular methods confirmed the presence and spread of the pathogen within Israel (Figs. 2 and 3). Arbitrarily primed PCR, which has been used extensively for genetic diversity studies of fungal and other populations proved reliable for this cause. In past studies we have used ap-PCR to determine clonality within populations of *Colletotrichum* species affecting almond, anemone, avocado and other hosts^30,31^, including species of *Fusarium* causing mango malformation disease^32^. Thus, it was no surprise that genetic uniformity existed among ap-PCR amplified DNA products from the 37 tested Israeli TR4 isolates compared to amplified fragments of four representative isolates from Jordan, the Philippines and Indonesia (Fig. 3).

PCR-specific primer amplification techniques are paramount for initial detection, identification and verification purposes of the presence of TR4. Numerous primers and PCR techniques (lamp, qPCR and regular PCR) have been used for detection purposes, some more effective than others^12,23,33,34^. For example, the primer set used by Lin *et al*.^34^ was not accurate for TR4 detection and cross reacted with other Foc race 1 isolates^33^. Likewise, the commonly used assay using specific primers of Dita *et al*.^23^ has been extensively used, however in some cases a false-negative reaction was evident in some of our tests (data not shown). Therefore, in this study we repeatedly used the diagnostic primers published by O’Neill *et al*.^24^. These primers successfully verified the identity of the tested TR4 isolates from Israel and representative DNA of TR4 isolates from other areas worldwide (Fig. 3).

Detection of TR4 from the Middle East was first confirmed in 2013 from Jordan^18^ although it was assumed that the pathogen had existed in that country since 2005^3^. At that time, no reports were available of TR4 infections from additional banana growing countries in the region. However, since the first report from Jordan, the pathogen has spread within that country^3^ (Freeman, personal comm.), has been detected from two locations in Lebanon^17^ and now is also well established in Israel^20,21^. The likelihood that the pathogen may be present in the Palestinian Authority (PA), within the territory where banana is cultivated in Jericho, should also be assessed, because extensive travel exists locally between the banana cultivation areas of the PA and Israel. Thus, it is imperative to determine the source or origin of the infections in Israel, in order to monitor and curb additional new introductions of the pathogen.

Recently, 251 single-nucleotide polymorphism (SNPs) were identified after sequencing 8 representative TR4 genomes allowing the clustering and subsequent phylogeographic relationship among isolates^8^. It was found that TR4 isolates from Vietnam, Laos, and Myanmar clustered with isolates from China (Yunnan province); isolates from Pakistan were closely related to those from the Philippines and those from Lebanon were genetically related to those from Jordan. Therefore, it can be postulated that the origins of related populations can be associated with that of the genetic relatedness using SNPs. In this study, we used SNP analyses of 5 representative TR4 isolates from the Philippines, Israel, Jordan and Indonesia, and compared them to those sequenced by Zheng *et al*.^8^ and by García-Bastidas *et al*.^16^. Isolates from Colombia clustered with the representative isolate from Indonesia, suggesting the source of incursion into South America. The Israeli isolates clustered with those from Jordan and Lebanon, indicating that the source of origin of the Israeli isolates is most likely from Jordan (Figs. 5 and 6), due to the fact that no overland transport exists between Israel and Lebanon. An additional asumption that the source of infection in Israel originated from Jordan, is due to the close proximity of infected plantations in Jordan to those in the Jordan Valley of Israel^3,18^, and possible movement of workers, soil, stray wild pigs and agricultural produce accross the borders. It is important to indicate that the SNP analyses did not indicate from where the initial infections of the Middle East originated, only the fact that the tested isolates within this geographical region were genetically similar.

In this report, we have updated our findings and data regarding the new incursions of Foc TR4 in the Middle East, specifically in Israel. This study further pronounces and stresses the gravity of the situation and the necessity to promote awareness campaigns in indicating the importance of TR4 within the banana industry community specifically, and the public in general. These efforts should also emphasize the priority of preventing local and international dissemination of TR4 isolates within the banana cultivation areas in Israel and from the cross-bordering infected plantation areas of Jordan.

## Methods

### Sample collection and fungal isolates

Commercial cultivar ‘Grande Naine’ Cavendish banana plants from Shfeya, Carmel coastal plain, and from Kibbutz Ein Gev, eastern shore of Lake Galilee, showing typical Fusarium wilt symptoms, accompanied by discoloration of the vasculature, mature leaf yellowing and dead leaves surrounding the pseudostem, were sampled during July and October 2016. Additional samples originated from a number of sites in close proximity to that of Ein Gev (Kibbutz Masada, Kibbutz Gesher and Kibbutz Deganya B), in the eastern/southern Lake Galilee area and Jordan Valley^21^ (Table 1).

The affected plant samples were processed for Foc TR4 isolation and characterization, as described^19^. Infected plant tissue was surface sterilized with 70% ethanol for 20 seconds, 1% sodium hypochlorite (NaOCl) for 3.5 minute, washed with sterile water and dried on sterilized tissue paper. The plant tissue was then placed aseptically on potato dextrose agar (PDA, Difco, USA) plates amended with 250 ppm chloramphenicol, and incubated at 25 °C. Cultures growing from affected tissue sections were further purified by the single spore method^35^. Representative Foc TR4 isolates from Israel and others used in this study, were kindly provided by R. Ploetz (Univ. of Florida, USA)^19^ and A. Viljoen (Stellenbosch Univ., South Africa)^36^ (Table 1). Subsequently, pathogenicity of representative TR4 isolates from Israel were verified, see below^19^.

### VCG characterization of isolates

Vegetative compatibility groupings (VCG) of representative isolates (Table 1) were conducted, as described^37^. Puhalla’s minimal nitrate agar (MM), a sucrose-salt medium containing nitrate as the nitrogen source^37,38^, was used to identify *nit* mutants and for complementation (heterokaryon) tests. Chlorate media, based on MM or potato dextrose agar amended with KClO_3_ (15 g/L), were used to generate *nit* mutants^36^. Plates (9-cm diam.) of chlorate media were centrally inoculated with 4-mm^3^ mycelial plugs and incubated at 25 ^o^C. Fast-growing sectors emerging from the restricted colonies were transferred to MM plates (5-cm diam.) and examined after a 4-day incubation period. Colonies with thin expansive mycelium were considered *nit* mutants. Complementation between *nit* mutants was tested on MM, as previously described^31^. Heterokaryons were usually evident within 10 to 15 days. When mutants formed a prototrophic heterokaryon, their parent isolates were assigned to the same VCG.

### DNA extraction and molecular analyses of isolates

Monoconidial cultures of 40 *F. o*. f. sp. *cubense* isolates (Table 1) were grown at 25 °C in liquid broth of glucose minimal medium, per liter composition: 50 ml of 20 × salt solution (120 gm of NaNO_3_, 10.4 gm of KCl, 10.4 gm of MgSO_4_.7H_2_O, 30.4 gm of KH_2_PO_4_ dissolved in one liter of distilled water), 1 ml of Hunter’s trace elements solution (2.2 gm of ZnSO_4_7H_2_O, 1.1 gm of H_3_BO_3,_ 0.5 gm of MnCl_2_4H_2_O, 0.5 gm of FeSO_4_7H_2_O, 0.16 gm of CoCl_2_5H_2_O, 0.16 gm of CuSO_4_5H_2_O, 0.11 gm of (NH_4_)6Mo_7_O_24_4H_2_, O.5 gm Na_4_EDTA dissolved in 100 ml of distilled water)^35^. Arbitrarily-primed (Ap)-PCR was performed on all the TR4 isolates using the following primers: (CAG)_5_, (GACA)_4_, (AGG)_5_ and (GACAC)_3_ ^30–32^. PCR reactions were conducted in 20 µl volume, containing 1.5 µl of total genomic DNA (100 ng/µl concentration), 2 µl of 10x Taq Buffer, 1 µl of 10 µM primer, 2 µl of 25 mM MgCl_2_, 2 µl of 10 mM dNTPs, 0.2 µl of Taq Polymerase enzyme and 11.3 µl of sterile water. PCR reactions were carried out in a thermocycler (Biometra, Germany) with the following cycling parameters: initial denaturation at 95 °C for 5 min, followed by 29 cycles of denaturation at 95 °C for 30 sec, annealing for 30 sec (60 °C for CAG_5_ and AGG_5_; 48 °C for GACAC_3_ and GACA_4_), and extension at 72 °C for 1.5 min, and a final extension at 72 °C for 15 min. PCR primers (TR4-F2 5′CAG GCC AGA GTG AAG GGG GAA T3′ and TR4-R1 5′CGC CAG GAC TGC CTC GTG A3′) were used for specific diagnostic amplification of a DNA fragment of 1400 bp for TR4 isolate detection^19,20,24^. PCR cycling parameters included initial denaturation at 95 °C for 10 min, followed by 35 cycles of denaturation at 95 °C for 30 sec, 68 °C for 90 sec and a final extension at 72 °C for 3 min. PCR amplification and the reaction results were kept at 4 °C until further processed. PCR products were then separated in 1.8% agarose gel (15 × 10 cm, W × L) in Tris-Acetate-EDTA buffer, at 80 V, 400 mA for 2 hours and stained with ethidium bromide (0.5 µg/ml) to visualize the banding patterns using ENDURO GDS gel documenting system (Labnet, USA). All PCR reactions were conducted at least three times with identical results.

### Pathogenicity assays

Three representative Israeli TR4 isolates (FOC TR4-1, FOC TR4-5 and FOC TR4-18 were used for plant (cv. ‘Grande Naine’) inoculation assays, as essentially described^19,20^. Conidia were produced from 1-week-old cultures grown in PDA dishes and plants with four true leaves were inoculated at a concentration of 10^6^ conidia/ml in 400 ml sterile water. Inoculation was conducted by dipping unwounded rooted plants for 30 min in conidial suspensions of the above isolates and water control. Inoculated plants were then planted in a soil mix (Green90; Evenari.co.il) in 750 ml pots under 30 °C and 16/8-h light/darkness photoperiod conditions in a quarantine greenhouse. Ten replicate plants were inoculated each with the TR4 isolates and water control. Experiments were repeated twice with similar results.

### Sequence analyses of Foc TR4 isolates

To determine the identity of the isolates and their relationship with other representatives, two TR4 isolates from Israel [FOC-TR4-1 (NRRL# 66915) from Shfeya and FOC TR4-5 (NRRL# 66916) from Ein Gev), one from Jordan (JV14), one from the Philippines (S1B8) and one from Indonesia (II-5) were arbitrarily selected for whole-genome sequencing at the Technion Genome Center, Haifa Israel, using Illumina technology (HiSeq. 2500), yielding ∼20 million cleaned reads (150 nt) (sequences were uploaded to SRA under BioProject accession PRJNA563197). An additional 8 representatives (JV11 from Jordan, Leb1.2 C from Lebanon, La-2 from Laos, My-1 from Myanmar, Vn-2 from Vietnam, Pak1-1A from Pakistan, Phi2-6C from the Philippines and II-5 from Indonesia), subsequently sequenced by Zheng *et al*.^8^, and 3 isolates (Col 2, Col 4 and Col 17 from Colombia) sequenced by García-Bastidas *et al*.^16^, were used for comparisons.

Single-nucleotide polymorphisms (SNPs) were identified using GATK version 3.4-0^39^ by mapping short reads against the Foc TR4 II5 reference isolate, using BWA-mem^40^. Duplicate reads were marked using Picard tools version 1.78 (http://broadinstitute.github.io/picard/). Genomic variants were identified using GATK HaploTypeCaller, and a joint variant call set was generated using GATK Genotype GVCFs. Subsequently, SNP variants were selected and filtered to retain high quality SNPs (only homozygote SNPs were selected), and used to determine the relationships between Foc TR4 isolates by way of principle component analyses TASSEL software^41^.

## Supplementary information

Supplementary Information.

Supplementary Information2.

## Data Availability

All genome sequence and SNPs data have been submitted to GenBank (sequences have been uploaded to SRA under BioProject accession PRJNA563197) and will be readily available upon acceptance of this manuscript. Representative TR4 isolates from Israel have been deposited in the USDA-ARS, NRRL culture collection, Peoria IL.
