# Supplementary material for: The origin and current situation of Fusarium oxysporum f. sp. cubense tropical race 4 in Israel and the Middle East
Source: Sci Rep. 2020 Jan 31;10:1590. doi: 10.1038/s41598-020-58378-9 (PMC6994609; doi:10.1038/s41598-020-58378-9)
Supplement: Supplementary file 1 — Supplementary Information. [file 41598_2020_58378_MOESM1_ESM.docx]

Marcel Maymon, Noa Sela, Uri Shpatz, Navot Galpaz & Stanley Freeman. The origin and current situation of *Fusarium oxysporum* f. sp. *cubense* tropical race 4 in Israel and the Middle East.

**Figure S1.**

**SIX1a**

**
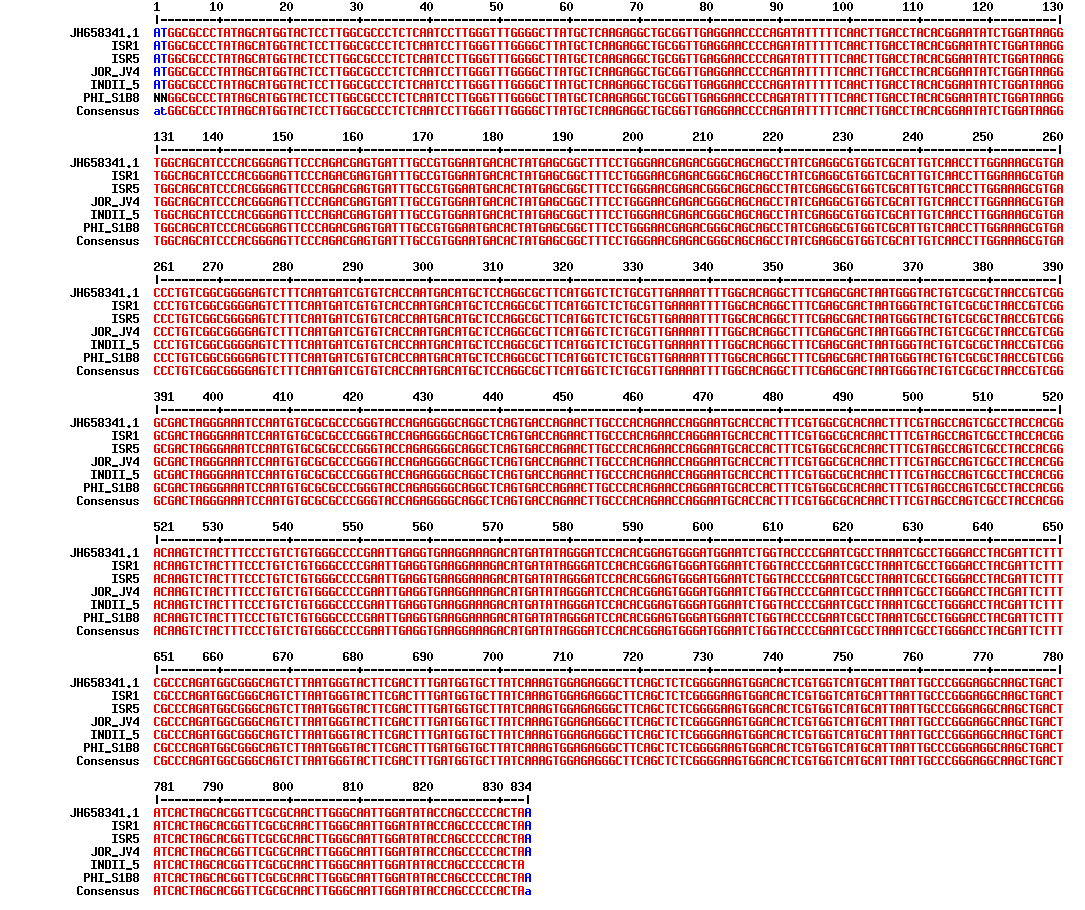
**

**SIX 1b**

**
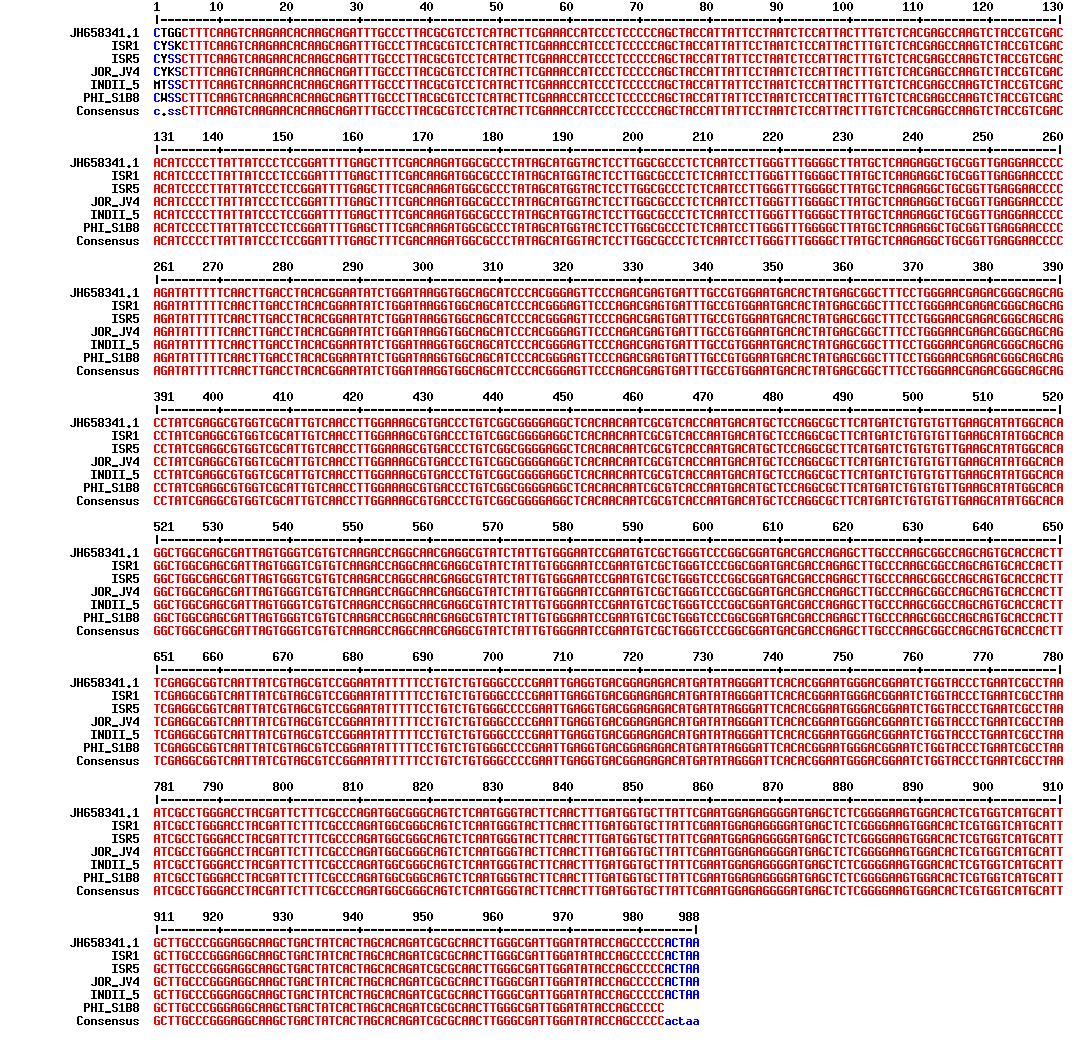
**

**SIX1c**

**
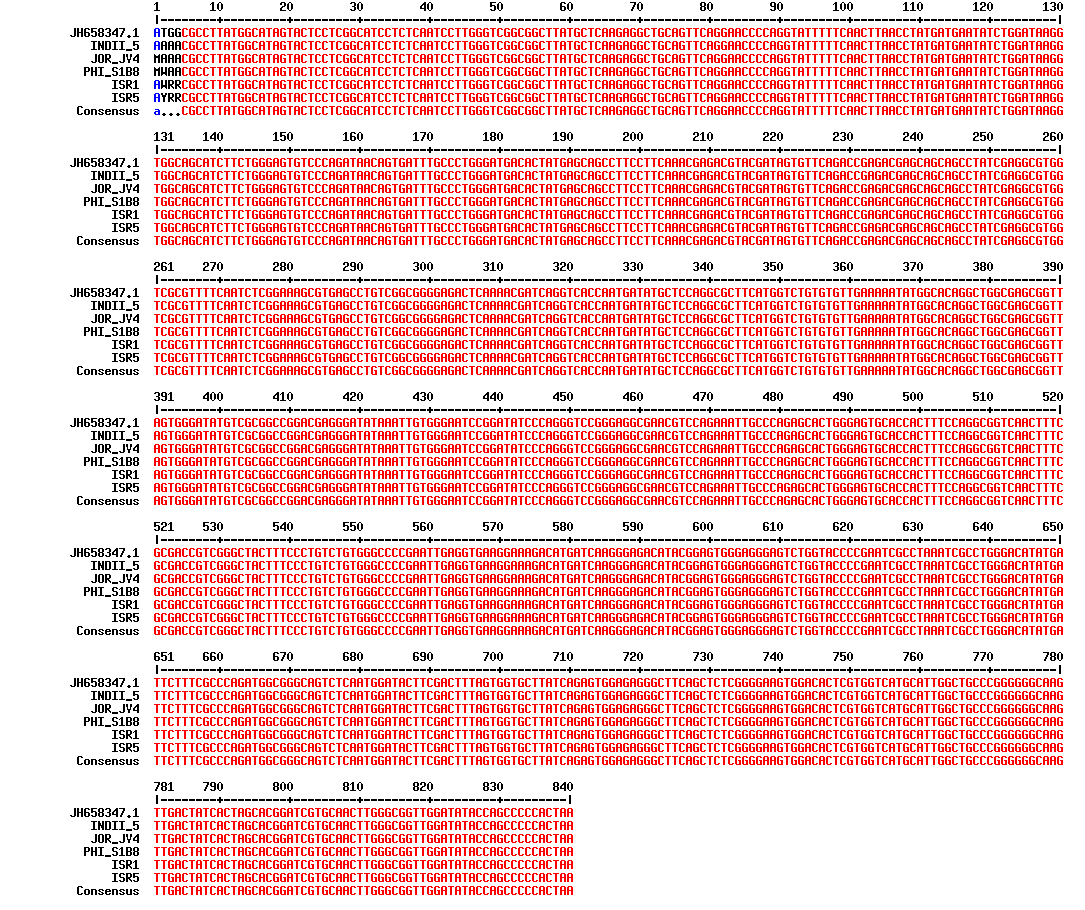
**

**SIX4**

**
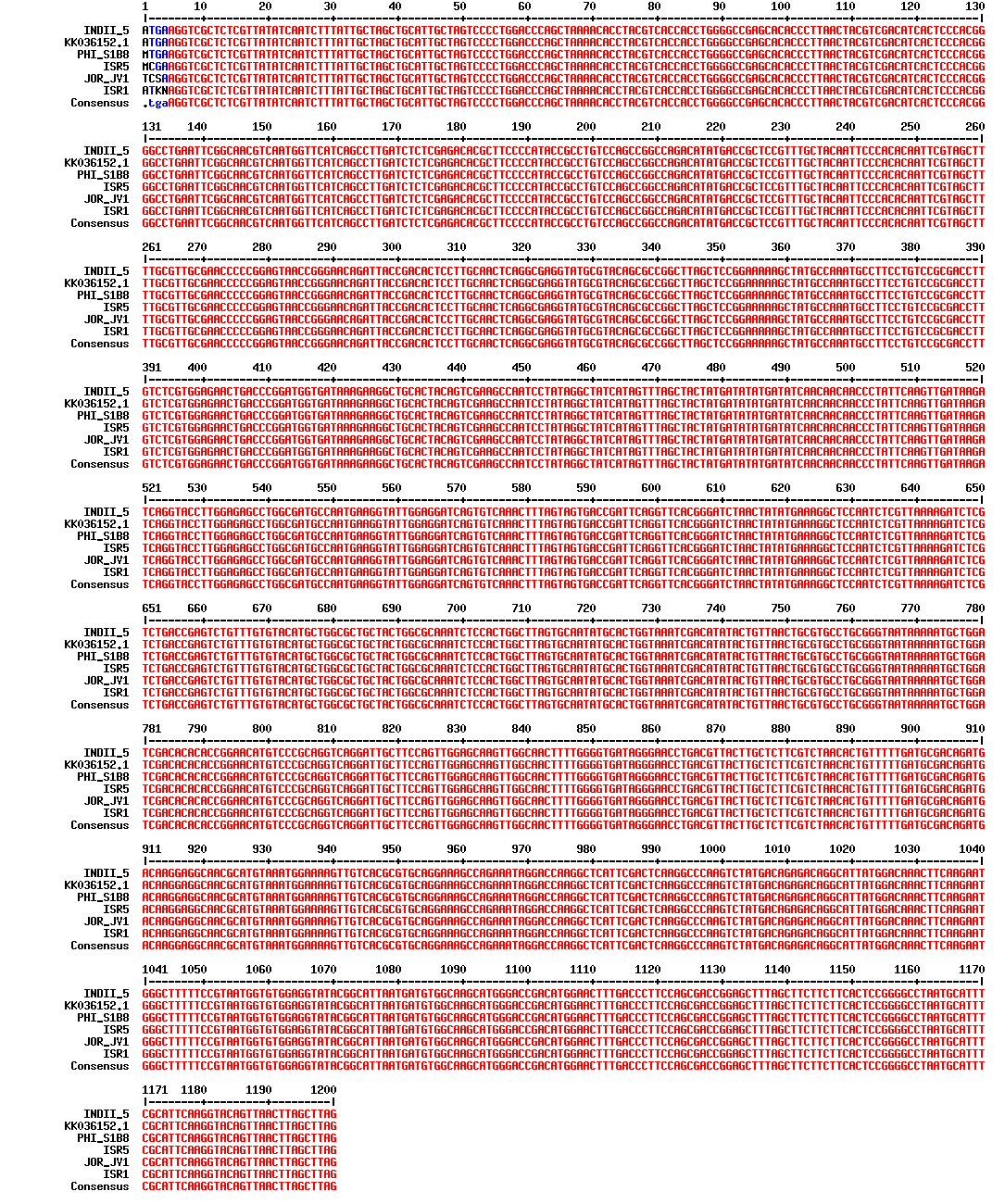
**

**SIX9**

**
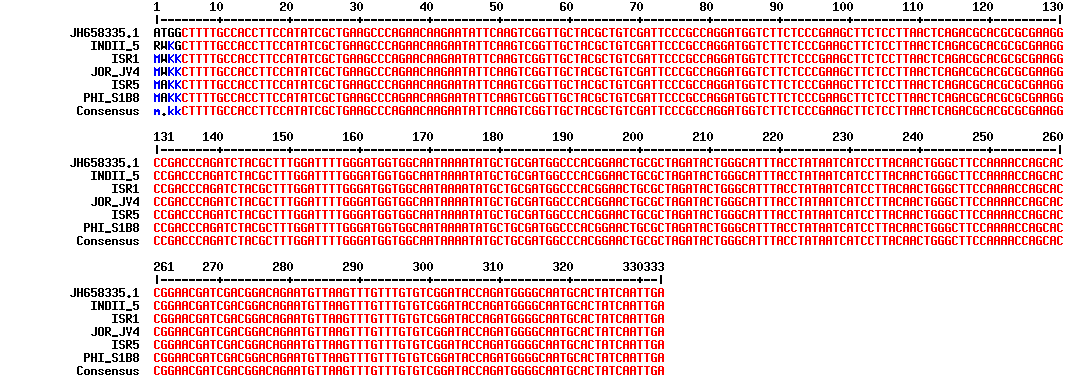
**

**Figure S1**.

SIX1a, SIX1b, SIX1c, SIX4, SIX9 gene homologues of TR4 isolates based on the consensus sequence calculated with SAM tools software [(Heng, L., et al. The sequence alignment/map format and SAMtools. Bioinformatics 25.16 (2009): 2078-2079]. The alignment shows no differences in sequences of the SIX genes between that of the NCBI downloaded accession (AGND00000000.1) and those of the different isolates sequenced in this study [JH658341.1 (reference NCBI strain); INDII_5 (II-5 from Indonesia); ISR1 (FOC-TR4-1 from Israel); JOR_JV4 (JV14 from Jordan); ISR5 (FOC TR4-5 from Israel) and PHI_S1B8 (S1B8 from the Philippines).
