# Supplementary figures and images for: The origin and current situation of Fusarium oxysporum f. sp. cubense tropical race 4 in Israel and the Middle East
Source: Sci Rep. 2020 Jan 31;10:1590. doi: 10.1038/s41598-020-58378-9 (PMC6994609; doi:10.1038/s41598-020-58378-9)

FIGURE 2


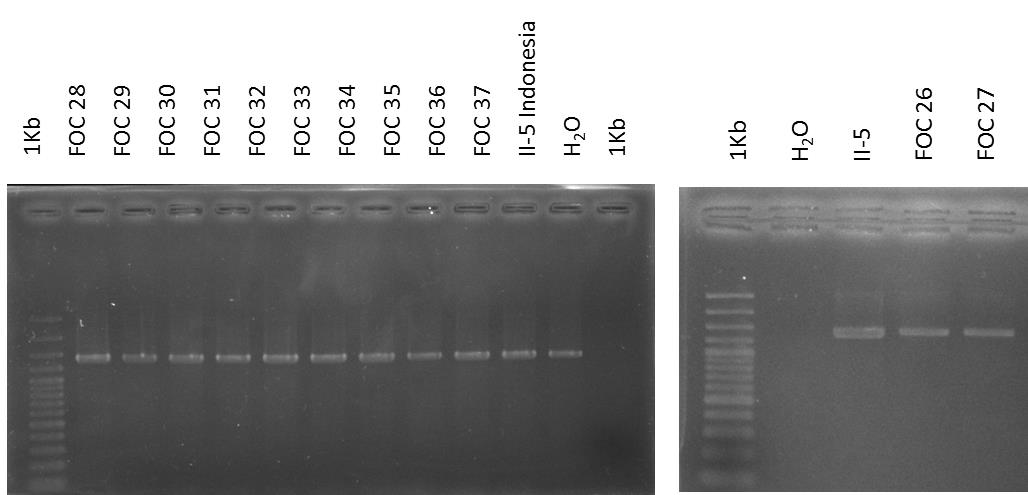


FIGURE 3


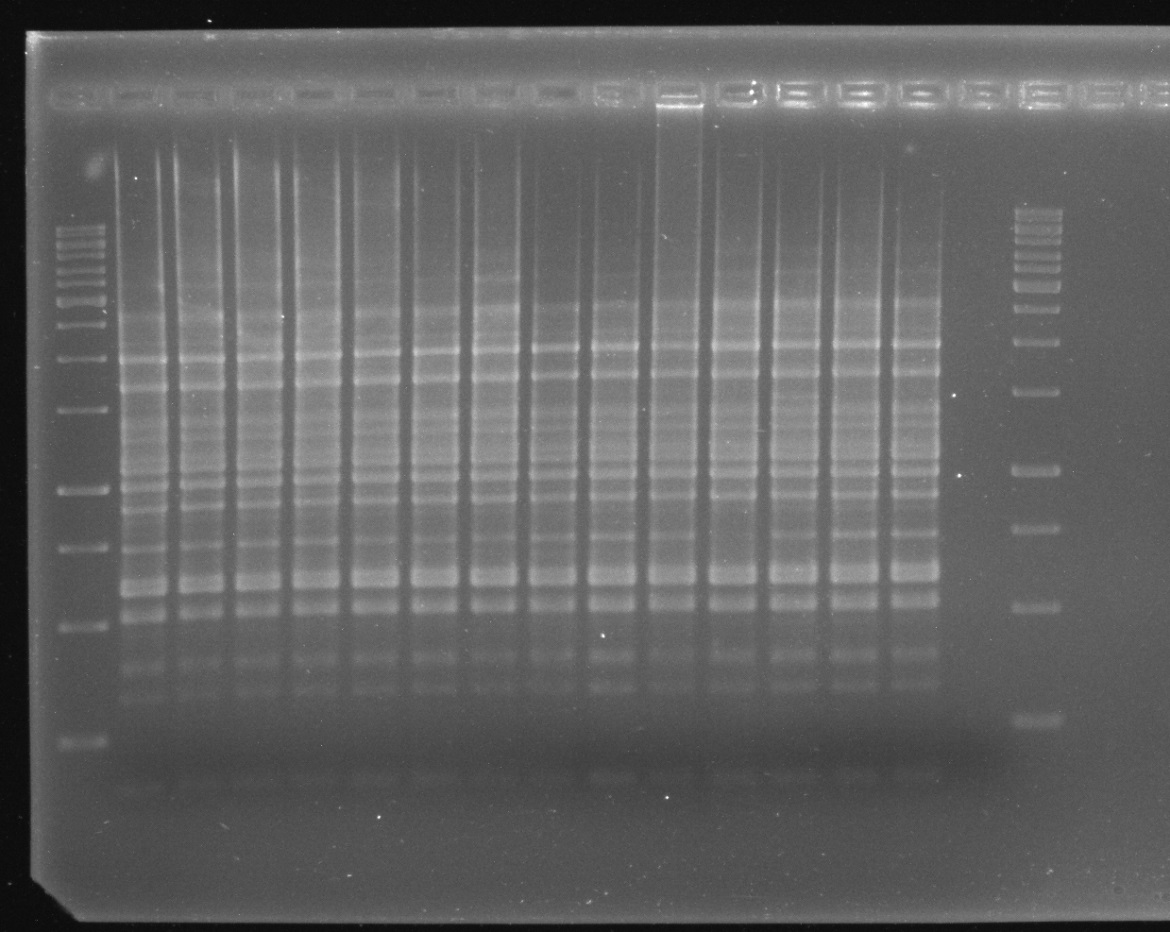

Supplement: Supplementary file 2 — Supplementary Information2. [file 41598_2020_58378_MOESM2_ESM.docx]
